# Vasorelaxant and Hypotensive Effects of Jaboticaba Fruit (*Myrciaria cauliflora*) Extract in Rats

**DOI:** 10.1155/2015/696135

**Published:** 2015-04-15

**Authors:** Daniela Medeiros Lobo de Andrade, Carolina de Fátima Reis, Patrícia Ferreira da Silva Castro, Leonardo Luiz Borges, Nathalia Oda Amaral, Ieda Maria Sapateiro Torres, Stefani Garcia Rezende, Eric de Souza Gil, Edemilson Cardoso da Conceição, Gustavo Rodrigues Pedrino, Matheus Lavorenti Rocha

**Affiliations:** ^1^Faculty of Pharmacy, Federal University of Goias, Rua 240, Esquina com 5^a^ Avenida, s/n, 74605-170 Goiânia, GO, Brazil; ^2^Center for Neuroscience and Cardiovascular Research, Biological Sciences Institute, Federal University of Goias, Campus Samambaia, 74001-970 Goiânia, GO, Brazil

## Abstract

This study's aim was to determine the effect of hydroalcoholic extract of *M. cauliflora* (HEMC) on vascular tension and blood pressure in rats. In our *in vitro* studies using precontracted isolated aortas from rats, HEMC and acetylcholine (positive control) induced relaxation only in vessels with endothelium. Pretreatment with L-NAME (NO synthase inhibitor) or ODQ (soluble guanylyl cyclase (sGC) inhibitor) abolished the HEMC-induced relaxation. The treatment with MDL-12,330A (adenylyl cyclase (AC) inhibitor) or diclofenac (COX inhibitor) reduced HEMC-induced vasorelaxation. The blockade of muscarinic and *β*-adrenergic receptors (by atropine and propranolol, resp.) did not promote changes in HEMC-induced vasorelaxation. In our *in vivo* studies, catheters were inserted into the right femoral vein and artery of anesthetized rats for HEMC infusion and the measurement of blood pressure, heart rate, and aortic blood flow. The intravenous infusion of HEMC produced hypotension and increased aortic blood flow with no changes in heart rate. These findings showed that HEMC induces endothelium-dependent vascular relaxation and hypotension with no alteration in heart rate. The NO/sGC/cGMP pathway seems to be the main cellular route involved in the vascular responsiveness.

## 1. Introduction

Jaboticaba is a fruit of the family Myrtaceae, with the most widespread varieties belonging to the genus* Myrciaria*. Amply distributed in the Brazil and South America, it is a globose fruit, with reddish bark that is nearly black, a whitish mucilaginous pulp, sweet to sour, and very tasty, which commonly presents a single seed. It is consumed* in natura*, in the form of liquor, jams, ice creams, juices, and alcoholic beverages, which have been increasing in consumption in Brazil and abroad [1, 2].

In addition of its use as food and beverage, folk medicine uses jaboticaba to treat asthma, throat inflammation, and gastrointestinal and cardiovascular disturbances [3]. Recent findings by Dragano et al. [4] and Araújo et al. [5] have shown that capacity to jaboticaba peels reduces significantly blood cholesterol (dyslipidemia) and obesity-associated insulin resistance, respectively.

The phytochemistry study of jaboticaba identified the presence of compounds including pyranocyanin B, quercetin, isoquercitrin, quercimeritrin, quercitrin, rutin, gallic acid, and ellagic acid, among others [6]. Some studies had shown the high antioxidant capacity of jaboticaba due to the presence of several compounds on different parts of the fruit. Moreover, higher concentration of phenolic compounds is present in the peel [7, 8].

The regulation of the vascular tone is very important to the appropriate control of blood pressure. In this way, some studies demonstrated that compounds obtained from medicinal plants in different forms present hypotensive and vasodilation effects that can contribute to the treatment of cardiovascular illnesses [9, 10].

Considering that the effects of this specimen on cardiovascular activity have not been explored yet, this study was performed with the aim to evaluate the cardiovascular property such as vasorelaxant effect, hypotensive, and hemodynamic parameters of jaboticaba fruit extract.

## 2. Materials and Methods

### 2.1. Extract Preparation

Jaboticaba fruit peels (*Myrciaria cauliflora *Berg) were donated by winery Jaboticabal in Hidrolândia, Goiás, Brazil (16° 57′ 57′′ S; 49° 13′ 35′′ W), in January 2012. A voucher specimen (number 21140) has been deposited in the herbarium of the ICB/UFG Botany Department. The peels were air dried, pulverized in a knife mill, and passed through a 60-mesh sieve in the Laboratory of Natural Products Research, Faculty of Pharmacy, Federal University of Goias. The powder obtained was stored at −20°C. To prepare the extract, 1 kg of the dried material was extracted by exhaustive percolation with ethanol : water (55 : 45 v/v) solution. The extract obtained was filtered and evaporated under reduced pressure on a rotatory evaporator at 40°C, obtaining 85.6 g of the hydroalcoholic extract of* Myrciaria cauliflora* (HEMC). After this process, the extract was kept in a freezer at −20°C without contact with the clarity and lighting. On the day of the experiments, HEMC was dissolved in distilled water to yield a 120 mg/mL solution.

### 2.2. Chemical Characterization of Extract

Total phenolic compounds were quantified in the HEMC according to Hagerman and Butler's method, adapted by Mole and Waterman [11]. For that, ferric chloride was added to an aqueous extract solution under alkaline conditions to result in a coloured complex with phenolic compounds (read at 510 nm). All solutions were prepared in triplicate. The standards curves were prepared with tannic acid at the dilutions 0.10, 0.15, 0.20, 0.25, and 0.30 mg/mL.

The extract was submitted to quantification of total tannins content employing Hagerman and Butler's method, adapted by Waterman and Mole [12]. The extract was precipitated with Bovine serum albumin (BSA) in 0.2 M acetate buffer (pH 4.9) and, after centrifugation, the precipitated (containing tannins) was dissolved in sodium dodecyl sulfate/triethanolamine solution, then ferric chloride was added and tannins were complexed (read at 510 nm). All solutions were prepared in triplicate. The standards curves were prepared with tannic acid at the dilutions 0.10, 0.20, 0.30, 0.40, and 0.50 mg/mL.

For phytochemical standardization of the HEMC, ellagic acid (determined by HPLC-PDA) was used as the chemical marker for this species [2, 13]. The HPLC analyses were carried out using a Waters LC system (Milford, Massachusetts, USA) comprising a quaternary pump, an on-line degasser, an autosampler, and a photodiode array detector model 2998. Empower 2.0 software was used for the control of the HPLC equipment and for the acquisition and treatment of data. Chromatographic separation was carried out with a C18 reverse phase column (250 × 4.6 mm, 5 *μ*m) purchased from Phenomenex (Phenomenex Inc., Torrance, CA, USA). The detection wavelength was 252 nm. The mobile phase was composed of methanol : water (60 : 40, v/v) at flow rate of 0.5 mL·min^−1^. The injection volume was set to 10 *μ*L, the temperature at 25°C and the run time at 10 min.

### 2.3. Animals

Male Wistar rats (200–250 g) from the central animal facility of the Federal University of Goiás were used in this study. All experiments were carried out in accordance with the Animal Research Ethical Committee of the Federal University of Goiás, Goiânia, GO, Brazil (protocol: 056/2013). This investigation conforms to EU Directive 2010/63/EU for the care and use of experimental animals.

#### 2.3.1. Preparation of Isolated Arteries

After euthanasia, the aorta were isolated and cut into rings approximately 4 mm long, placed between two stainless-steel stirrups, and connected to a computerized system and a WinDaq Resource (DATAQ Instruments, Akron, OH, USA) data acquisition unit to measure isometric tension in the preparations. The aortic rings were placed in a 10 mL organ chamber containing Krebs solution with the following composition: 130 mM NaCl, 4.7 mM KCl, 1.2 mM KH_2_PO_4_, 1.2 mM MgSO_4_, 14.9 mM NaHCO_3_, 5.5 mM glucose, and 1.6 mM CaCl_2_. The solution was maintained at pH 7.4 and gassed with 95% O_2_ and 5% CO_2_ at 37°C. The rings were initially stretched to a basal tension of 1.0 g. In some preparations, the endothelium was mechanically removed and the removal's effectiveness was demonstrated by the absence of relaxation to acetylcholine (1 *μ*M) in aortic rings precontracted with phenylephrine (0.1 *μ*M).

To investigate the mechanism(s) responsible for HEMC-induced relaxation, aortic rings were contracted with phenylephrine (0.1 *μ*M) 30 minutes after incubation with the following drugs: (1) the NO synthase (NOS) inhibitor L-NAME (100 *μ*M); (2) the nonselective COX inhibitor diclofenac sodium (10 *μ*M); (3) the association L-NAME (100 *μ*M) plus diclofenac sodium (10 *μ*M); (4) the selective soluble guanylyl cyclase (sGC) inhibitor 1H-[1,2, 4]oxadiazolo-[4,3-a]quinoxalin-1-one (ODQ, 1 *μ*M); and (5) the selective adenylyl cyclase (AC) inhibitor cis-N-(2-phenylcyclopentyl)-azacyclotridec-1-en-2-amine hydrochloride (MDL-12,330A, 10 *μ*M). To assess the involvement of muscarinic receptors or *β*-adrenergic receptors in the relaxation response evoked by HEMC, the experiments were carried out after incubation (20 min) with atropine (1 *μ*M) or propranolol (10 *μ*M).

#### 2.3.2. Measurement of Hemodynamic Parameters (*In Vivo* Experiments)

On the day of the experiments, rats were anesthetized with halothane (2-3% halothane in 100% O_2_), and catheters were inserted into the right femoral vein and artery. After catheter placement, the rats were removed from the halothane, and anesthesia was maintained by intravenous administration of urethane (1.2 mg/kg body weight; Sigma-Aldrich Co., St. Louis, MO, USA). In the experiments that measured aortic blood flow (ABF), miniature ultrasonic transit-time flow probes (Transonic Systems Inc., Ithaca, NY, USA) were placed around the aorta. Body temperature was kept at 37 ± 0.5°C with thermostatically controlled heated table. To measure blood pressure, the arterial catheter was connected to a pressure transducer attached to abridge amplifier (ETH-200; CB Sciences, Dover, NH, USA). Pulsatile pressure was recorded continuously with an analog-to-digital converter (PowerLab System, ADInstruments, Colorado Springs, CO, USA). Mean arterial pressure (MAP) and heart rate (HR) were calculated from the pulsatile signal using Chart software (version 7.3.1; ADInstruments, Colorado Springs, CO, USA). To measure aortic blood flow (ABF), a flow probe was connected to an ultrasonic transit-time flow meter, as described before [14, 15] (Transonic Systems Inc., Ithaca, NY, USA). Either extract (0.012, 0.12, 0.24, 0.48, and 0.96 mg/kg, b.wt.), vehicle (Saline, 0.9% NaCl), or sodium nitroprusside (control group) (0.02 mg/kg b.wt.) was infused (i.v. in 0.1 mL) through the femoral vein cannula.

### 2.4. Reagents

All chemicals of reagent grade were obtained from Sigma (Sigma-Aldrich Inc., St. Louis, MO, USA). All other chemicals used in the present study were commercially available and of reagent grade. The purity of all substances was >98%. The concentrations given are as final concentrations in the bath solution.

### 2.5. Statistical Analysis

The results are expressed as mean ± SEM. The maximum effect (*E*_max⁡_) was considered as the maximal amplitude response reached in the concentration-effect curves. The IC_50_ (the concentration to produce a 50% of the maximal contraction in response to phenylephrine) value was determined from the concentration-response curve by linear interpolation (HEMC 0–120 *μ*g/mL). Statistical two-way repeated-measures ANOVA and Bonferroni posttest data evaluations were performed using the program Prism 5.0, GraphPad (Software Inc., San Diego, CA, USA). Values of *P* < 0.05 were considered to be significant.

## 3. Results

### 3.1. Chemical Characterization of Extract

The HEMC showed 17.89% of total phenolic compounds, calculated on the total solids content in the extract. It was not possible to quantify the total tannins content employing the protein precipitation assay due the very low absorbance, even with the extract not diluted.

**Figure 1:**
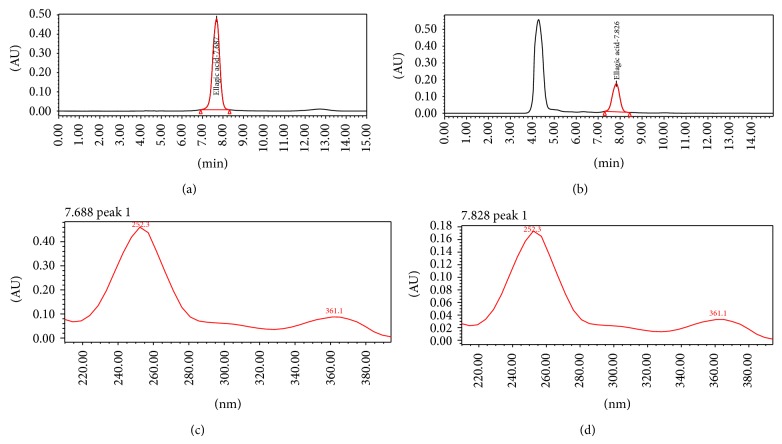
Chromatogram of standard of ellagic acid (a) and sample of hydroalcoholic extract of* Myrciaria cauliflora* (HEMC) (b). UV-spectra corresponding to ellagic standard (c) and sample of hydroalcoholic extract of* Myrciaria cauliflora* (HEMC) (d).

**Figure 2:**
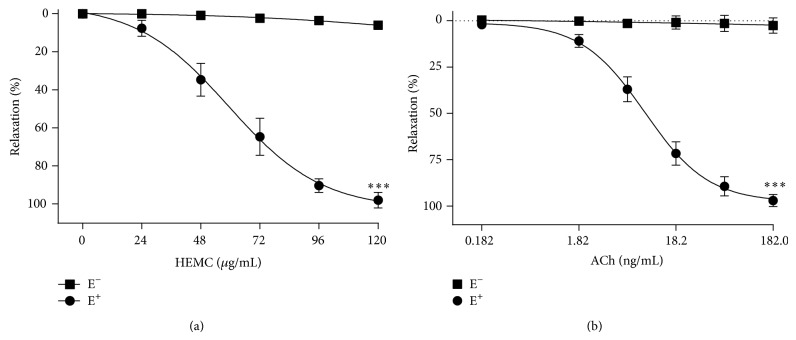
The relaxant effect of the HEMC in isolated rat thoracic artery. Cumulative concentration-response curves for the HEMC (a) or acetylcholine (Ach) (b), a well-known endothelium-dependent vasorelaxant (positive control), in rings of thoracic artery with endothelium (E^+^, *n* = 6) and without endothelium (E^−^, *n* = 6) precontracted with phenylephrine (0.1 *μ*M). The data points represent the mean ± SEM of the relaxing effect expressed as a percentage. Significant difference ^∗∗∗^ *P* < 0.001 (E^+^ versus E^−^).

In order to analyze the extract used in this study, HPLC was performed. The HEMC showed the presence of ellagic acid (Rt: 7.826 min). Compounds were identified by comparison with external standard (HPLC chromatograms not shown). The level of ellagic acid (chemical marker) found in HEMC was 0.2221% (w/w). Figures 1 and 2 show the chromatograms (Figures 1(a) and 1(b)) and UV-spectra (Figures 1(c) and 1(d)) regarding standard of ellagic acid and the analyzed extract, which confirm the presence of this marker in the sample evaluated.

### 3.2. Vascular Effects of HEMC

The HEMC elicited a concentration-dependent relaxant effect in vessels containing a functional endothelium (E^+^), reaching an *E*_max⁡_ = 99.5 ± 1.4% (*n* = 6) at an HEMC concentration of 120 *μ*g/mL (Figure 2). The IC_50_ value for the HEMC in preparations E^+^ was 61.8 ± 6.9 *μ*g/mL (Table 1). The vasorelaxation effect of HEMC was abolished in the absence of a functional endothelium (E^−^) (*E*_max⁡_ = 4.1 ± 0.9%, *n* = 6), as shown in Figure 2 and Table 1, indicating that the vasodilator effect of HEMC depended on endothelium-derived relaxing factors.

**Figure 3:**
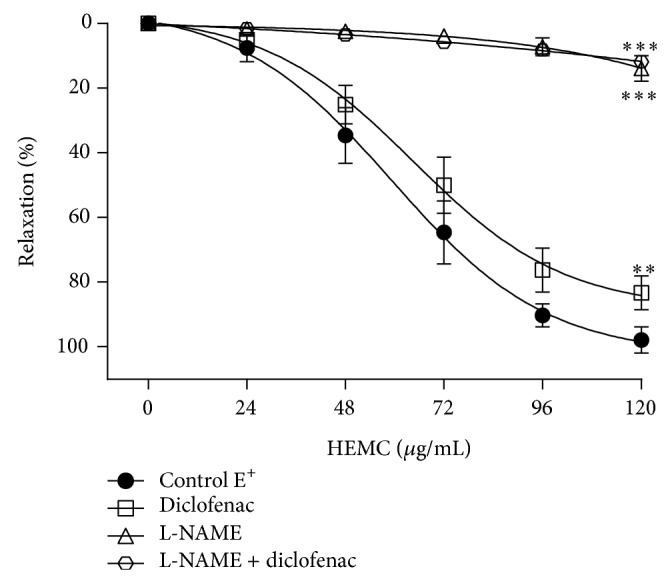
Effect of diclofenac, L-NAME, and L-NAME + diclofenac on relaxation of isolated rings of thoracic artery induced by HEMC. Cumulative concentration-response curves for the HEMC before and after incubation (30 min) with diclofenac (10 *μ*M, *n* = 7) or L-NAME (100 *μ*M, *n* = 8) or L-NAME + diclofenac (100 *μ*M + 10 *μ*M, *n* = 5) in rings with functional endothelium precontracted with phenylephrine (0.1 *μ*M). The data points represent the mean ± SEM of the relaxing effect expressed as a percentage. Significant difference ^∗∗^ *P* < 0.01; ^∗∗∗^ *P* < 0.001 compared to control.

The NOS inhibitor L-NAME and the COX inhibitor diclofenac sodium significantly reduced the *E*_max⁡_ for the HEMC from 99.5 ± 1.4% to 13.8 ± 3.6% (*n* = 8, *P* < 0.001) and 84.5 ± 4.6% (*n* = 7, *P* < 0.01), respectively (Figure 3; Table 1). When incubating the two inhibitors simultaneously, the effect was similar to the results obtained previously with L-NAME (11.9 ± 1.8%, *n* = 5; Figure 3; Table 1). The IC_50_ values for the HEMC in preparations E^+^ in the presence or absence of diclofenac were similar (Table 1). The inhibitory effect of L-NAME was greater than diclofenac, suggesting that relaxation induced by the HEMC exhibits superior dependence on the NOS pathway.

**Figure 4:**
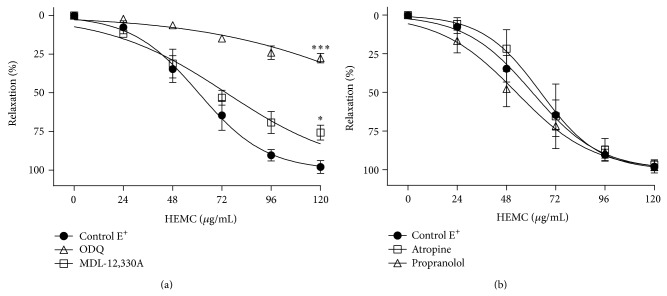
The relaxant effect of the HEMC in isolated rat aorta precontracted with phenylephrine. (a) Before and after incubation (30 min) with MDL-12,330A (10 *μ*M, *n* = 5) or ODQ (1 *μ*M, *n* = 6) in rings with functional endothelium. (b) Before and after incubation (30 min) with atropine (1 *μ*M, *n* = 6) or propranolol (10 *μ*M, *n* = 6) in rings with functional endothelium. The data points represent the mean ± SEM of the relaxing effect expressed as a percentage. Significant difference ^∗^ *P* < 0.05; ^∗∗∗^ *P* < 0.001 compared to control.

The AC inhibitor MDL-12,330A and the sGC inhibitor ODQ significantly reduced the *E*_max⁡_ for the HEMC from 99.5 ± 1.4% to 77.8% ± 5.8 (*n* = 5) and 32.7 ± 3.8 (*n* = 6), respectively (Figure 4(a); Table 1). The IC_50_ values for the HEMC in preparations E^+^ in the presence or absence of MDL-12,330A were similar. This value was elevated in preparations treated with ODQ (Table 1). The inhibitory effect of ODQ was greater (*P* < 0.001) than that of MDL-12,330A, suggesting that relaxation induced by the HEMC depends more on the sGC/cGMP pathway than the AC/cAMP pathway.

The *β*-blocker propranolol and the cholinergic receptor antagonist atropine did not show any change in the profile relaxation caused by the HEMC (Figure 4(b); Table 1), demonstrating that there is no involvement at the level of these receptors in the profile relaxation induced by the HEMC.

### 3.3. Hemodynamic Alterations Induced by HEMC

**Figure 5:**
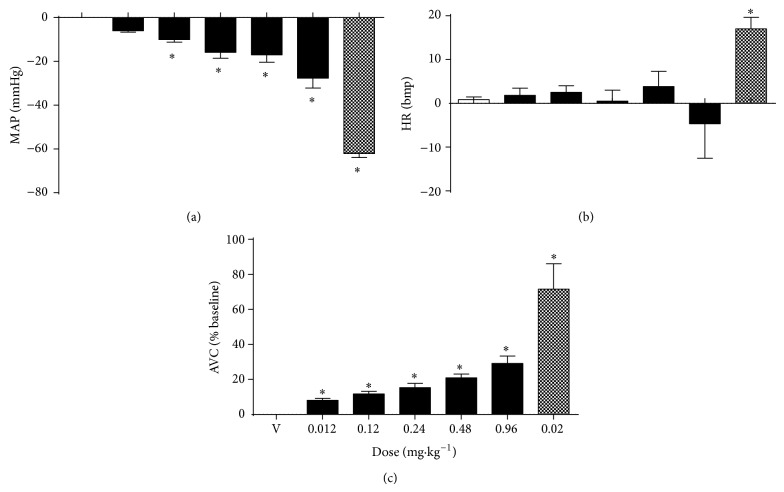
Effects of HEMC infusion (0.012, 0.12, 0.24, 0.48, and 0.96 mg/kg; black bars), vehicle infusion (V; 0.9% Saline; white bars), or sodium nitroprusside (positive control; 0.02 mg/kg; gray bars) on cardiovascular parameters in anesthetized rats. (a) Mean arterial pressure (MAP). (b) Heart rate (HR). (c) Aortic vascular conductance (AVC). The results are expressed as the mean ± SEM of 6 experiments. ^∗^ *P* < 0.05 compared to vehicle (V).

In anesthetized rats (*n* = 6), infusion of the vehicle did not promote changes in MAP, HR, or AVC (Figures 5(a), 5(b), and 5(c)). Intravenous infusion of an HEMC produced significant, dose-dependent hypotension and aortic vasodilation. At doses of 0.12, 0.24, 0.48, and 0.96 mg/kg, b.wt., treatment with an HEMC decreased MAP (−10 ± 1.2 mmHg; −16 ± 2.7 mmHg; −17 ± 3.4 mmHg; −28 ± 4.5 mmHg from the baseline value, resp.; Figure 5(a)), did not promote changes in HR (3 ± 1.5 bpm; 1 ± 2.5 bpm; 4 ± 3.5 bpm; −5 ± 7.9 bpm from the baseline value, resp.; Figure 5(b)), and increased AVC (12 ± 1.5%; 15 ± 2.5%; 21 ± 2.1%; 29 ± 4.4% from the baseline value, resp.; Figure 5(c)). At dose of 0.02 mg/kg, intravenous infusion of sodium nitroprusside (internal control) induced hypotension (−62 ± 1.8 mmHg; Figure 5(a)) and increased HR (17 ± 2.7 bpm; Figure 5(b)) and AVC (71 ± 14.5% from the baseline value; Figure 5(c)).

## 4. Discussion 

The major findings of this study are that extract obtained from jaboticaba induced hypotension associated with an increased aortic vascular conductance without promoting changes in heart rate. The extract also evoked relaxation in isolated arteries with functional endothelium, revealing a significant dependence of the endothelial cells on vascular effects of the extract in the tested concentrations. These results show that Jaboticaba contains substance(s) that may interfere with the function of endothelial vascular cells and dose dependently reduce the blood pressure without cardiac effects.

The short-term systemic blood pressure control in humans and mammals is carried out by a sophisticated multi-input and output, multifeedback system involving hormonal and neural regulations that exert strong control on vessels and the heart. Thus, one substance that interferes with the functionality of vessels or heart will change the blood pressure quickly [16]. In this study, the infusion of HEMC in rats led to the fall of blood pressure and increase of AVC, indicating aortic vasodilation. In addition, we did not observe alterations in the heart rate, indicating an absence of cardiac effects of HEMC. Therefore, the hypotensive effects observed after HEMC infusion are likely caused by systemic vasodilation.

Regarding phytochemical characterization, it is known that during food processing ellagitannins change to free ellagic acid and its derivatives [17]. Therefore, ellagic acid has a great efficiency as antioxidant compound due the presence of several hydroxy groups, which are responsible for the strong potential to donate a hydrogen atom and support the unpaired electron [18]. The antioxidant activity decreases with the presence of a sugar moiety; thus the free ellagic acid has a greater activity when compared with its bound forms [19]. Also, the main ellagitannin metabolites circulating in plasma are ellagic acid, which are released upon hydrolysis and under the physiological conditions of the gastrointestinal tract [20].

According to Abe et al. [2], the content of free ellagic acid in fruits of* M. cauliflora* varied from 0.00028% to 0.0085% (w/w) and total ellagic acid content from 0.0215% to 0.311% (w/w). The level of ellagic acid found in HEMC was 0.2221% (w/w), so the concentration of this compound in residues is in agreement with contents in fruits. There has been an increasing interest in this chemical marker due to its powerful antioxidant activities and other properties such as cardioprotective effects, inhibition of the growth of some human pathogenic bacteria, inhibition of *α*-amylase and angiotensin I-converting enzymes (ACE), and antiproliferative activities against several different cancer cell lines [20, 21].

It has been accepted that a diet rich in fruits and vegetables offers protection against common diseases such as cancer and several cardiovascular illnesses. This evidence encourages a search for the identification of phytochemicals that express advantageous biological effects. The natural compounds that have been studied widely are the phenolic compounds in plant foods as their intake has been associated with reduced damages in the cardiovascular system [6, 10]. Some studies showed a high antioxidant capacity of jaboticaba due to the presence of several phenolic compounds in the fruit peels [8, 13, 22]. This study showed a high concentration of phenolic compounds in the HEMC. Due to the association to phenolic compounds and cardiovascular effects shown in this study, jaboticaba consumption can be an important ally to preventing or treating circulatory system diseases.

The endothelial cells can modulate the vascular smooth muscle response to different contractile or relaxant stimuli [23]. The complete loss of vascular response to the HEMC after endothelium denudation indicates that the vasorelaxant activity of the HEMC is endothelium dependent. Additionally, we considered that HEMC could stimulate both the endothelium-dependent NO and the prostanoids pathway. Prostaglandin I_2_, produced by COX, is a well-known vasodilator [23, 24], acting via activation of the adenylate cyclase system and increasing cAMP and protein kinase A activity among other effects [24]. NO and COX products were found to mediate that effect because exposure of the aortic rings to L-NAME and diclofenac sodium inhibited the relaxant response to the HEMC. However, inhibition of NO synthase is more critical than COX inhibition.

Increases in cytosolic levels of cyclic nucleotides (cAMP and cGMP) cause vasodilation by activating protein kinases A (PKA) and G (PKG). This activation induces many cellular effects that reduce intracellular Ca^2+^ levels, leading to relaxation [25, 26]. This study showed that ODQ and MDL-12,330A (an inhibitor of sGC and AC, resp.) significantly reduced the HEMC-induced relaxation. These results implied that the vasorelaxation induced by HEMC involved the AC/cAMP and GCs/cGMP pathways, indicating that this vasorelaxant response is at least partly dependent on the activation of the cyclic nucleotides. Moreover, the inhibition of NO/sGC/cGMP pathways (with L-NAME and ODQ) was more efficient in inhibiting relaxation, revealing a greater dependence of this pathway.

We also investigated the mechanism involved in the ability of HEMC to stimulate nitric oxide release. Under physiological conditions, nitric oxide is continuously released by the NO synthase in endothelial cells as a consequence of the shear stress generated by blood flow. However, its release is also mediated by activation of muscarinic [27] and *β*-adrenergic [28] receptors, among others. This agrees with our data showing that atropine (a muscarinic receptor antagonist) and propranolol (a *β*-adrenoceptor antagonist) cannot impair the vasodilatory action of HEMC. Therefore it is conceivable to suggest that HEMC evokes stimulus in NO/sGC/cGMP and AC/cAMP pathways independent of muscarinic and *β*-adrenergic receptor activation.

## 5. Conclusion

This study, which used both* in vivo* and* in vitro* approaches, demonstrates that extract of jaboticaba fruit contains high concentration of phenol compounds and induces hypotension associated and vascular relaxation dependent on the endothelium in a low concentration (120 *μ*g/mL). The NO/sGC/cGMP pathway seems to be the main cellular route involved in the relaxation induced by HEMC. Therefore, the biological effects induced by jaboticaba shown in our work will contribute to the body of knowledge about jaboticaba-derived compounds and their use as medicinal plant allied for cardiovascular illnesses prevention and health.

## Figures and Tables

**Table 1:** Effect of different inhibitors/antagonists on the relaxation induced by HEMC of rat aortic rings precontracted with phenylephrine.

Groups	*E*_max⁡_ (% Relaxation)	IC_50_ (*µ*g/mL)
Control E^−^	4.1 ± 0.9 (6)^***^	nd
Control E^+^	99.5 ± 1.4 (6)	61.8 ± 6.9
L-NAME	13.8 ± 3.6 (8)^***^	nd
Diclofenac	84.5 ± 4.6 (7)^**^	63.8 ± 5.1
L-NAME + diclof.	11.9 ± 1.8 (5)^***^	nd
ODQ	32.7 ± 3.8 (6)^***^	78.7 ± 6.3^*^
MDL-12,330A	77.8 ± 5.8 (5)^*^	62.7 ± 4.8
Atropine	96.7 ± 3.8 (6)	62.8 ± 6.2
Propranolol	98.2 ± 4.9 (6)	58.5 ± 5.9

Number between parentheses indicates the number of animals. Values are means ± S.E.M.

Asterisks compared to group E^+^ (ANOVA followed by Newman-Keuls multiple comparison test, ^*^ *P* < 0.05; ^**^ *P* < 0.01; ^***^ *P* < 0.001 (nd = not determined due to the intense inhibitory effect)).
